# Lab Scale Extracted Conditions of Polyphenols from Thinned Peach Fruit Have Antioxidant, Hypoglycemic, and Hypolipidemic Properties

**DOI:** 10.3390/foods11010099

**Published:** 2021-12-31

**Authors:** Kun Dai, Yingying Wei, Shu Jiang, Feng Xu, Hongfei Wang, Xin Zhang, Xingfeng Shao

**Affiliations:** Zhejiang-Malaysia Joint Research Laboratory for Agricultural Product Processing and Nutrition, College of Food and Pharmaceutical Sciences, Ningbo University, Ningbo 315800, China; 15957178580@163.com (K.D.); weiyingying@nbu.edu.cn (Y.W.); jiangshu@nbu.edu.cn (S.J.); xufeng1@nbu.edu.cn (F.X.); wanghongfei@nbu.edu.cn (H.W.); zhangxin@nbu.edu.cn (X.Z.)

**Keywords:** thinned peach, polyphenols, UPLC-ESI-QTOF-MS/MS, antioxidation, hypoglycemic, hypolipidemic

## Abstract

Thinned peach polyphenols (TPPs) were extracted by ultrasonic disruption and purified using macroporous resin. Optimized extraction conditions resulted in a TPPs yield of 1.59 ± 0.02 mg GAE/g FW, and optimized purification conditions resulted in a purity of 43.86% with NKA-9 resin. TPPs composition was analyzed by UPLC-ESI-QTOF-MS/MS; chlorogenic acid, catechin, and neochlorogenic acid were the most abundant compounds in thinned peaches. Purified TPPs exhibited scavenging activity on DPPH, ABTS, hydroxyl radical, and FRAP. TPPs inhibited α-amylase and α-glucosidase by competitive and noncompetitive reversible inhibition, respectively. TPPs also exhibited a higher binding capacity for bile acids than cholestyramine. In summary, TPPs from thinned peaches are potentially valuable because of their high antioxidant, hypoglycemic, and hypolipidemic capacities, and present a new incentive for the comprehensive utilization of thinned peach fruit.

## 1. Introduction

In recent years, agricultural by-products have attracted increasing attention because of their potential value as raw materials [1]. Horticultural crops with high fruit setting rates, such as apples, pears, grapes, and peaches, are often thinned to ensure fruit yield and quality [2]. During thinning, large quantities of immature fruits are routinely discarded; the yield of thinned fruits per acre is 30–50 kg/acre for thinned apples, and 100 kg/acre for thinned pears and thinned peaches [2]. Unfortunately, thinned fruit is typically regarded as a nearly worthless by-product rather than as a potentially valuable raw material. While some thinned fruit is used as poultry feed, most of what remains is abandoned in the field, where it causes great harm to soil and plants [3].

Thinned fruit is rich in polyphenols that possess antioxidant, antibacterial, and anticancer activities [2,4]. Polyphenol concentrations in thinned apple are approximately 10 times higher than in mature apples, and the antioxidant levels decrease gradually as the fruit reaches maturity [5]. Sun et al. [6] showed that the composition of polyphenols in thinned and mature pears differ significantly. The most abundant polyphenolic constituents in thinned fruit are chlorogenic acid, quinic acid, arbutin, and ursolic acid, which are the primary contributors to antioxidant capacity. Karabiyikli and Oncul [7] found that the thinned grape verjuice has antibacterial effect on foodborne pathogens, and the inhibitory effect is closely related to the content of polyphenols. Nasser et al. [4] reported that an extract prepared from thinned grapes exhibits high antioxidant capacity and significantly inhibits the viability of human pulmonary adenocarcinoma cells.

Studies on thinned peaches have focused on the quantity, composition, and antioxidant activity of polyphenols in crude extracts. Redondo et al. [8] found that concentrations of phenol and flavonoid in peaches decrease from thinning to harvest, and also showed that chlorogenic acid and neochlorogenic acid are abundant in thinned peaches. Guo et al. [9] showed that thinned peach extracts have a higher antioxidant capacity than those obtained from mature peaches, and the antioxidant capacity is significantly correlated with endogenous compounds including phenols, polysaccharides, organic acids, and amino acids. However, few studies have explored methods for the extraction and purification of thinned peach polyphenols (TPPs). At the same time, some studies have shown that the biological activity of purified polyphenols is significantly stronger than that of crude polyphenols [10]. In addition, other biological properties of interest, such as the hypoglycemic and hypolipidemic activities of TPPs, have also not been surveyed thoroughly.

The aim of this study is to develop extraction and purification methods for polyphenols from thinned peaches, and to evaluate extract composition and biological activity. We first optimized ultrasound-assisted protocols for extracting polyphenols from thinned peaches. Then, TPPs were purified using macroporous adsorption resin, and characterized and identified by UPLC-ESI-QTOF-MS/MS. Finally, the antioxidant, hypoglycemic, and hypolipidemic activities of TPPs were evaluated. Our results provide the basis for the development of methods for processing thinned peach fruits and recovering valuable compounds from them.

## 2. Materials and Methods

### 2.1. Plant Materials

Thinned unripe peaches (*Prunus persica* L. cv. Hujingmilu) were hand-harvested 25 day after blossoming from an orchard in Fenghua, Ningbo, Zhejiang Province, China. The fruits were transferred to our laboratory, selected, washed, cut, then quick-frozen in liquid nitrogen and pulverized to a uniform powder using a liquid nitrogen grinder (A11 basic, IKA, Staufen, Germany). Pulverized fruit were stored at −80 °C until use.

### 2.2. Optimization of Conditions for Ultrasonic Assisted Extraction of TPPs

#### 2.2.1. Single Factor Experiments

The single factors were as follows: ultrasonic times were 10, 20, 30, 40, and 50 min. Ultrasonic temperatures were 30, 40, 50, 60, and 70 °C. Ultrasonic power levels were 60, 90, 120, 150, and 180 W. Solid-to-liquid ratios were 1:6, 1:8, 1:10, 1:12, and 1:15 g/mL. Total phenolic content was determined by Mokrani and Madani [11]. The results were described as mg gallic acid equivalents per gram on a fresh weight basis (mg GAE/g FW).

#### 2.2.2. Response Surface Design Experiments

On the basis of the results of single factor experiments, a four-factor and three-level response surface experiment was designed using Design-Expert 8.0 (Stat-Ease, Inc., Minneapolis, MN, USA), and the experiment is based on the design method of Box–Behnken. The experimental design conditions are shown in Supplementary Materials Table S1.

### 2.3. Purification of TPPs by Macroporous Adsorption Resin

#### 2.3.1. Screening of Macroporous Resins

The physical characterization of four selected resins were described in Table S2. Before use, resins were pretreated as depicted by others [12]. 1 g of pretreated resin was mixed with 40 mL of 0.15 mg/mL TPPs, and then incubated in a rotary shaker (ZQZY-78BN, Shanghai Zhichu Instrument Factory, Ltd., Shanghai, China) for 24 h at 120 rpm and 25 °C. After reaching adsorption equilibrium, resins were cleaned with ultrapure water and desorbed by incubation in 100 mL of 70% ethanol under the same conditions described.

Adsorption and desorption rates were calculated using the equations [12]:

$$\mathrm{Adsorption}\;\mathrm{rate}=\frac{C_{0}-C_{e}}{C_{0}}\times 100\%$$

$$\mathrm{Desorption}\;\mathrm{rate}=\frac{C_{d}V_{d}}{(C_{0}-C_{e})V_{t}}\times 100\%$$

where *C*_0_ is the original concentration of polyphenols (mg/mL), *C_e_* is the equilibrium concentration of polyphenols (mg/mL), *C_d_* is the desorption concentration of polyphenols (mg/mL), *V_d_* is desorption volumes (mL), and *V_t_* is TPPs volume (mL).

#### 2.3.2. Static Adsorption and Desorption Kinetic Curves for NKA-9 Resin

The static adsorption and desorption conditions of NKA-9 were the same as the above screening process. Total phenolic content was measured at 0, 1, 2, 3, 4, 5, 6, 7, 8, 9, 10, 12, and 24 h, then the adsorption and desorption kinetic curves for NKA-9 resin were drawn.

#### 2.3.3. Dynamic Adsorption and Desorption Curves under Optimal Conditions

Dynamic adsorption and desorption tests were completed as depicted by Jiao et al. [13]. Briefly, a 10 × 100 mm column was loaded with NKA-9 resin for a bed volume (BV) of 5 mL. The effects of pH (2, 3, 5, 7, 8), sample concentration (0.2, 0.5, 1.0, 1.5, 2.0 mg/mL), adsorption flow rate (0.5, 1.0, 1.5, 2.0, 2.5, 3.0 mL/min), ethanol concentration (30, 50, 70, 90, 100%), and desorption flow rate (0.5, 1.0, 1.5, 2.0, 2.5, 3.0 mL/min) on dynamic adsorption/desorption behaviors were analyzed. After completion of adsorption and desorption, the total polyphenol concentration in each sample was determined, and the adsorption and desorption rates were calculated.

At the optimal adsorption and desorption conditions, the effluent was gathered at intervals of 0.5 BV to measure polyphenol content, and the dynamic adsorption/desorption curve for the NKA-9 resin was determined. Finally, the purified polyphenols were concentrated under vacuum, freeze dried (Scientz-10ND, Ningbo Xinzhi Instrument Factory, Ltd., Ningbo, China), and stored at −80 °C for subsequent experiments.

### 2.4. Identification and Quantification of TPPs by the UPLC-ESI-QTOF-MS/MS

Samples were analyzed by UPLC-ESI-QTOF-MS/MS (Agilent 6545 Q-TOF) matched with a COSMOSIL 5C18-MS-II column (4.6 × 250 mm, 5 μm) as referenced by Ding et al. [14] with minor modifications. Mass spectrometry data were obtained using the positive ion mode, and the molecular scanning size was 10–3000 m/z. The ionization conditions were as follows: gas temperature was 320 °C, gas flow was 8 L/min, and the nozzle voltage was 1 kV. The UPLC conditions were as follows: mobile phase A was water (1% formic acid), and mobile phase B was acetonitrile (1% formic acid). The gradient eluted program was as follows: solvent B: 5% (0–2 min) → 45% (16 min) → 90% (18 min) → 90% (22 min) → 5% (22.1 min) → 5% (26 min). The column temperature was 40 °C, the flow speed was 0.7 mL/min, the injection volume was 5 μL. The polyphenols were quantified by UV-DAD detector and the detection wavelength was 280 nm. At the same time, the standard curves were used to analyze the content of the main polyphenols (Table S3). The MassHunter application was used for data acquisition and processing.

### 2.5. Antioxidant Capacity of TPPs

#### 2.5.1. 2,2-Diphenyl-1-picrylhydrazyl (DPPH) Radical Scavenging Capacity

The DPPH radical scavenging capacity of the TPPs was determined according to Sun et al. [6]. Briefly, 0.4 mL of TPPs (at concentrations of 24, 48, 72, 96, and 120 μg/mL) was blended with 4 mL 100 μM DPPH ethanol solution. The mixture was shaken violently and reacted in the avoid light environment for 30 min at 37 °C, then absorbance was determined at 517 nm. Ethanol as a negative control and ascorbic acid as a positive control (at the same concentration as the sample). All measurements were performed in triplicate and the DPPH radical scavenging rate was measured as follows:

$$\mathrm{DPPH}\;\mathrm{radical}\;\mathrm{scavenging}\;\mathrm{rate}=\frac{A_{0}-A_{1}}{A_{0}}\times 100\%$$

where *A*_0_ is the absorbance of the ethanol, and *A*_1_ is the absorbance of sample.

#### 2.5.2. 2,2′-Azino-di-3-ethylbenzthiazoline Sulfonic Acid (ABTS) Radical Scavenging Effect

ABTS radical scavenging effect was determined as depicted by Liu et al. [15] with minor adjustments. Briefly, the determination solution was fabricated by mixing 7 mM ABTS and 2.45 mM potassium persulfate (K_2_S_2_O_8_) and reacting at ambient temperature for 16 h. 0.2 mL of sample (at 24, 48, 72, 96, and 120 μg/mL) was mixed with 4 mL of the diluted determination solution, and incubated at 37 °C for 6 min in the dark, and absorbance was detected at 734 nm.

#### 2.5.3. Hydroxyl Radical Scavenging Capacity

Hydroxyl radical scavenging capacity was determined as described by Ge et al. [16]. Briefly, 2 mL of sample (24, 48, 72, 96, and 120 μg/mL) was mixed with 0.5 mL of 9 mM FeSO_4_·7H_2_O and 0.5 mL of 8.8 mM H_2_O_2_, then incubated at 37 °C for 30 min, and absorbance was detected at 510 nm.

#### 2.5.4. Ferric Reducing Antioxidant Power (FRAP)

FRAP was performed as described by Ge et al. [16] with minor adjustments. Briefly, the FRAP was obtained by mixing 1:1:10 (*v*/*v*/*v*) 10 mM TPTZ in 40 mM HCl, 20 mM FeCl_3_·6H_2_O, and 0.3 M acetate buffer (pH 3.6). 200 μL of sample (24, 48, 72, 96, and 120 μg/mL) was mixed with 3.6 mL FRAP solution, and reacted at 37 °C for 10 min. Absorbance was detected at 593 nm. Ethanol was used the negative control, and ascorbic acid (at the same concentration as the sample) was used as the positive control.

### 2.6. Hypoglycemic and Hypolipidemic Effects of TPPs In Vitro

#### 2.6.1. α-Amylase Inhibition by TPPs

The α-amylase inhibition rate was determined as described by Fei et al. [17] with modifications. Briefly, 200 μL of TPPs (0.1, 0.5, 1, 3, and 5 mg/mL) was mixed with 200 μL 0.1 mg/mL α-amylase, and incubated at 37 °C for 5 min. 0.5 mL of 1% (*w*/*v*) starch substrate solution was added and the solution was reacted again at 37 °C for 4 min. 0.5 mL of 3,5-dinitrosalicylic acid (DNS) was supplemented and the solution was reacted at 100 °C for 8 min to stop the reaction. Absorbance was detected at 540 nm. Ultrapure water as negative control and acarbose (at the same concentration as the TPPs) as positive control.

The inhibitory activity of TPPs against α-amylase was measured using a fixed substrate concentration of 0.5% and TPPs concentration of 0.5 mg/mL. A curve was plotted with enzyme concentration (0.1, 0.5, 1, 3, and 5 mg/mL) on the *x*-axis and velocity on the *y*-axis. To further investigate TPPs inhibitory properties, α-amylase was reacted with various concentrations of substrate (2, 5, 8, and 10 mg/mL). Specific inhibition characteristics were determined using Lineweaver–Burk double reciprocal plots.

#### 2.6.2. α-Glucosidase Inhibition by TPPs

The α-glucosidase inhibition rate was determined as described by Wang et al. [18] with modifications. Briefly, 500 μL of 0.2 U/mL α-glucosidase was incubated with 200 μL of TPPs (concentrations at 10, 20, 50, 80, and 100 μg/mL) at 37 °C for 5 min. Then, the reaction was activated by adding 0.5 mL of 2.5 mM 4-nitrophenyl-α-D-glucopyranoside (PNPG) substrate solution, followed by incubation at 37 °C for 15 min. The reaction was stopped by addition of 1 mL 0.2 M Na_2_CO_3_. Absorbance was detected at 405 nm. Moreover, the α-glucosidase inhibition rate and inhibition type were referred to α-amylase methods.

#### 2.6.3. Determination of Bile Acid Binding Activity of TPPs

The binding activity was performed as described in Chen et al. [19] with sodium taurocholate and sodium glycocholate used as bile acids. Briefly, 2 mL of 0.2 mg/mL sodium taurocholate/sodium glycocholate in 0.1 M phosphate buffer, pH 7.2, were mixed with TPPs at 1, 2, 5, 8, and 10 mg/mL and incubated at 37 °C for 1 h. The mixture was centrifuged at 8000 rpm for 20 min. Then, 1 mL supernatant was mixed with 3 mL of 60% sulfuric acid and incubated at 70 °C for 20 min. Absorbance was detected at 387 nm. The bile acid binding activity was determined using a cholestyramine standard curve (Figure S1).

### 2.7. Statistical Analysis

Data are expressed as means ± standard deviation (SD), Differences were analyzed for significance using one-way-analysis (ANOVA) and Duncan’s multiple range test. Computations were analyzed using SPSS 25.0.

## 3. Results

### 3.1. Influence of Single Factor on TPPs Yield

**Figure 1:**
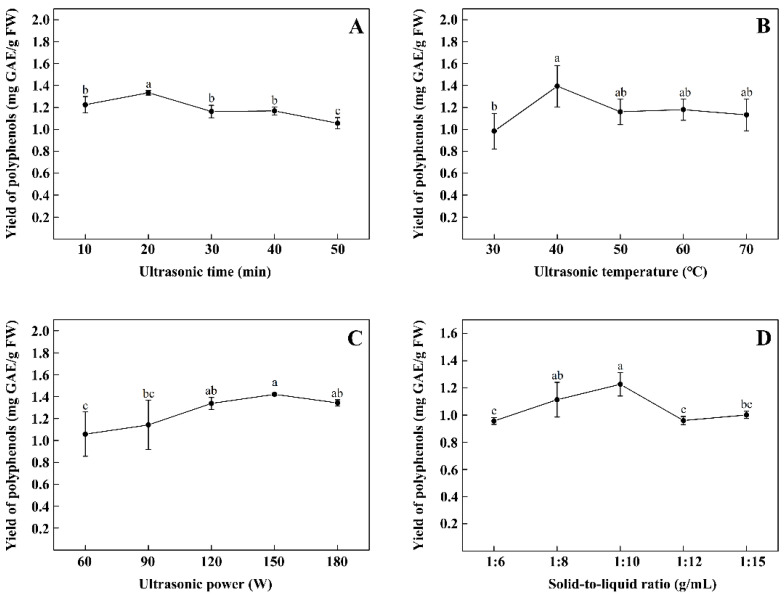
Effect of different extraction parameters on yield of polyphenols from thinned peach. Ultrasonic time (**A**), ultrasonic temperature (**B**), ultrasonic power (**C**), and solid-to-liquid ratio (**D**). Different letters indicate that there are significant differences (*p* < 0.05).

Figure 1 shows the results of the tested extraction parameters on TPPs yield. Yield increased with ultrasonic treatment up to 20 min (*p* < 0.05), then decreased thereafter (*p* < 0.05) (Figure 1A). Yield increased with increasing temperature during ultrasonic treatment up to 40 °C (*p* < 0.05), then further increased the temperature, the yield of polyphenols had no significant effect (*p* > 0.05) (Figure 1B). Yield increased with ultrasonic power up of 150 W (*p* < 0.05, except 120 W), and then continued to expand the gradient, which had no significant effect on the yield (*p* > 0.05) (Figure 1C). Yield increased as the solid-to-liquid ratio increased up to 1:10 g/mL (*p* < 0.05, except 1:8 g/mL) then decreased thereafter (*p* < 0.05) (Figure 1D). Thus, the inflection points chosen for the following response surface model were as follows: ultrasonic time (20 min), ultrasonic temperature (40 °C), ultrasonic power (150 W), and solid-to-liquid ratio (1:10 g/mL).

### 3.2. Optimization of Extraction Conditions Using a Response Surface Model

Response surface data were analyzed using the Design-Export application and the results are shown in Table 1. The yield of TPPs ranged from 1.066 to 1.547 mg GAE/g FW. Multiple regression was used to determine the relationship between the independent variables and the yield of polyphenols, with the following result:

Y = 1.41 − 0.015 A + 0.059 B + 0.013 C + 0.18 D + 0.054 AB − 0.046 AC + 0.025 AD − 0.026 BC − 0.021 BD + 0.018 CD − 0.069 A^2^ − 0.03 B^2^ − 0.099 C^2^ − 0.046 D^2^.

**Figure 2:**
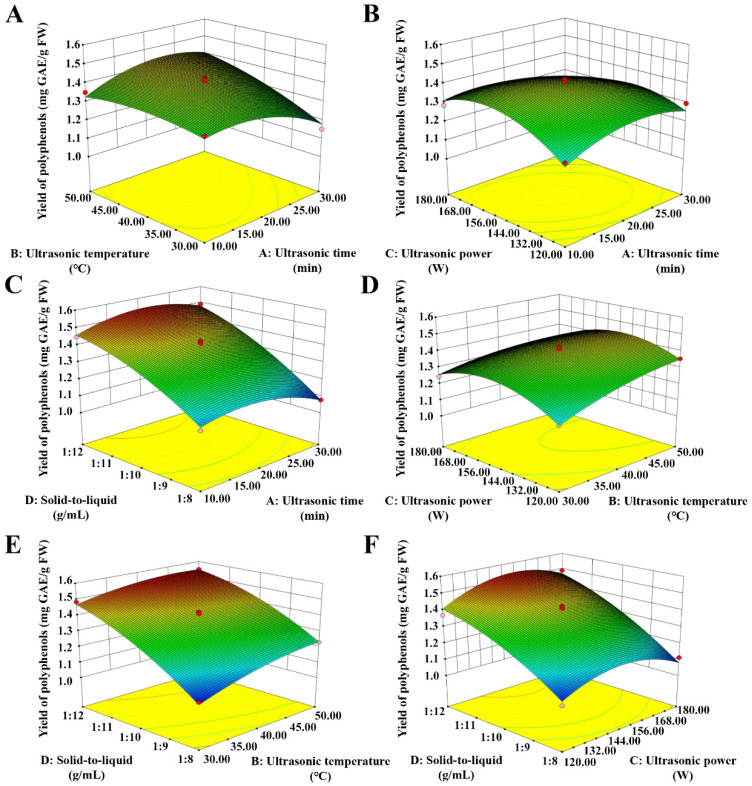
Response surface 3D plots showing combined effects of extraction parameters on yield of polyphenols from thinned peach. (**A**) Ultrasonic temperature and ultrasonic time; (**B**) ultrasonic power and ultrasonic temperature; (**C**) ultrasonic power and ultrasonic time; (**D**) solid-to-liquid ratio and ultrasonic temperature; (**E**) solid-to-liquid ratio and ultrasonic time; (**F**) solid-to-liquid ratio and ultrasonic power.

The analysis of variance of the response surface revealed that the model was extremely significant (*p* < 0.0001), the lack of fit was not significant (*p* > 0.05), the correlation coefficient R^2^ was 0.9816, and the adjusted determination coefficients R_adj_^2^ was 0.9632 (Table 2). These results demonstrate that the model can be used to predict the yield of polyphenols. The *p* values indicate the degree of influence each factor had on TPPs yield. The results show that the effects of solid-to-liquid and ultrasonic temperature on the yield were extremely significant (*p* < 0.0001), followed by ultrasonic time (*p* = 0.0757), and then ultrasonic power (*p* = 0.1152). In addition, from the response surface 3D plots (Figure 2C,E,F), it can be seen that for each time, temperature or power, the yield is the highest when the solid-to-liquid reaches the maximum, indicating that the solid/liquid ratio is the most important factor affecting the yield. Similarly, there is interaction between ultrasonic temperature and ultrasonic time (*p* = 0.0011) (Figure 2A). It can be seen that if the temperature is too low, increasing time has no significant effect on the yield, and if the temperature is too high, increasing time may lead to the degradation of polyphenols and decrease the yield. It is worth noting that although there is no significant effect on ultrasonic power (*p* > 0.05), there is interaction between ultrasonic power and ultrasound time (*p* = 0.0039) (Figure 2B). Similar to the interaction between ultrasonic temperature and ultrasonic time, the increase in power and time leads to the degradation of polyphenols. The optimum ultrasonic extraction conditions proposed by the model: ultrasonic time of 25 min, ultrasonic temperature of 50 °C, ultrasonic power of 147 W, and a solid-to-liquid ratio of 1:12 g/mL. After validation, the yield of polyphenols was 1.59 ± 0.02 mg GAE/g FW (*n* = 3), which was 98.5% of the predicted value.

### 3.3. Screening of Macroporous Resins, and Static Adsorption and Desorption Kinetic for NKA-9

**Figure 3:**
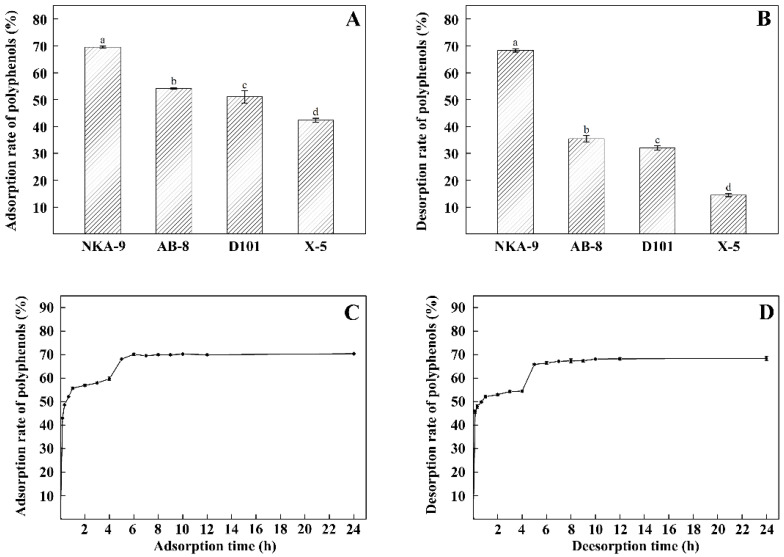
Absorption (**A**) and desorption (**B**) capacity of different resins; static adsorption (**C**) and desorption (**D**) kinetic curves of NKA-9. Different letters indicate that there are significant differences (*p* < 0.05).

The capacity of the tested resins to adsorb/desorb TPPs decreased in the order: NKA-9 > AB-8 > D101 > X-5 (Figure 3A,B). The adsorption/desorption rates of NKA-9 were the highest of the four resins at 69.53% and 68.30%, respectively. Therefore, NKA-9 was selected for use in subsequent experiments. The static adsorption and desorption kinetic for NKA-9 revealed that the adsorption and desorption rates of NKA-9 resin increased over time up to 6 and 5 h, respectively, at which time the processes reached equilibrium (Figure 3C,D).

### 3.4. Dynamic Adsorption and Desorption Curves under Optimal Conditions

**Figure 4:**
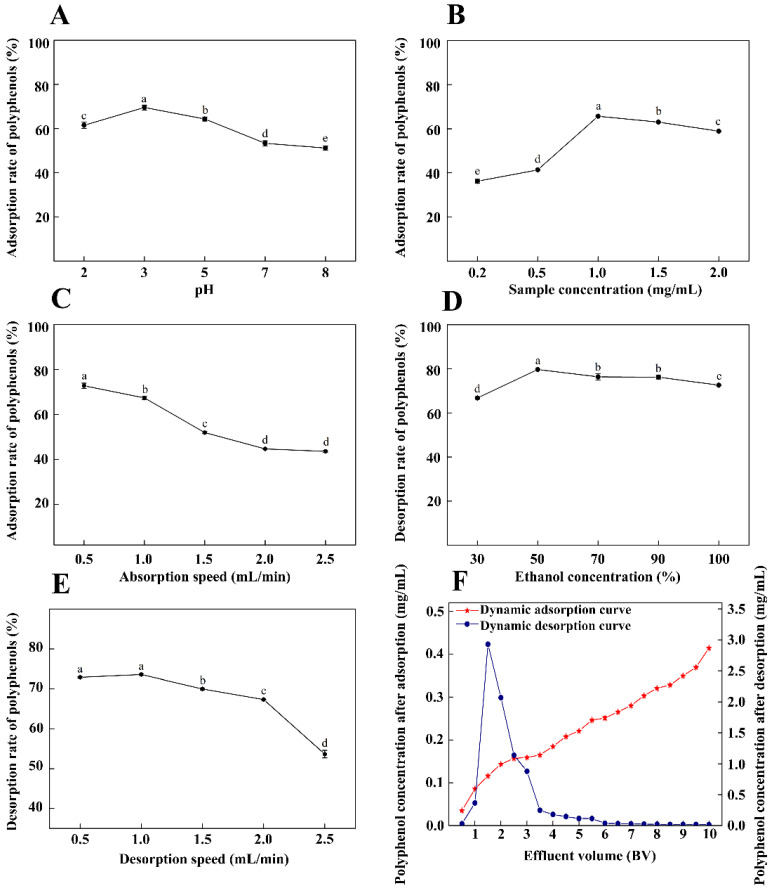
Effects of different factors on adsorption and desorption: pH (**A**), sample concentration (**B**), adsorption speed (**C**), ethanol concentration (**D**), and desorption speed (**E**). Dynamic adsorption and desorption curves under optimal conditions (**F**). Different letters indicate that there are significant differences (*p* < 0.05).

The effects of different factors on the adsorption and desorption rate of TPPs is shown in Figure 4. The adsorption rate of the NKA-9 resin was best at pH 3, decreasing thereafter with increasing pH (Figure 4A). The adsorption rate increased with increasing sample concentration up to 1.0 mg/mL, then decreased thereafter with increasing concentration (Figure 4B). The adsorption effect improved as flow speed decreased, and at speeds lower than 1.0 mL/min, the result was more than 80% (Figure 4C). The desorption rate was highest (79.67%) using a 50% ethanol solution, but the rate did not decrease substantially with increasing ethanol concentration (Figure 4D). The effect of desorption speed was similar to that of adsorption speed, such that the highest desorption rate was obtained when the desorption speed was 1.0 mL/min (Figure 4E).

At optimal conditions for adsorption and desorption, when the loading volume was 1.5 BV, the polyphenol concentration was one tenth of the initial polyphenol concentration (Figure 4F), indicating that adsorption had reached a leak point. The desorption curve shows that the elution peak was narrow. Polyphenols were primarily concentrated in the effluent of 1.5–3.5 BV, and desorption equilibrium was reached at an effluent volume of 6 BV. At the optimal loading (1.5 BV) and elution (6 BV) volumes, purity increased from 1.93% to 43.86%.

### 3.5. Identification and Quantification of TPPs by UPLC-ESI-QTOF-MS/MS

**Figure 5:**
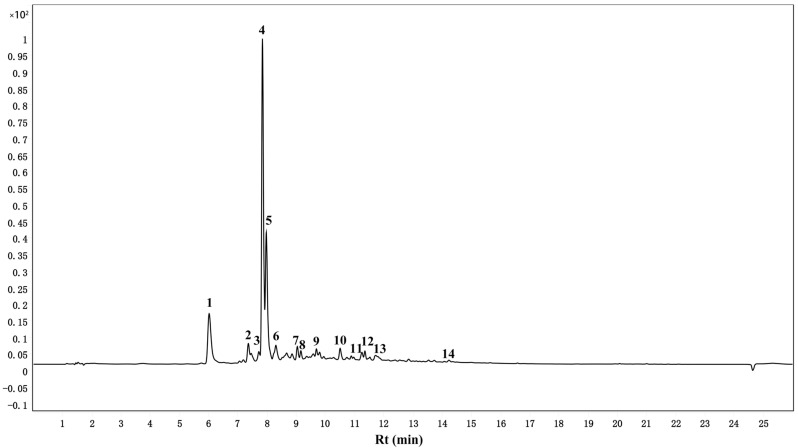
UPLC-ESI-Q-TOF-MS/MS chromatograms of TPPs: (1) Neochlorogenic acid; (2) Cyanidin-3-glucoside; (3) Procyanidin B1; (4) Chlorogenic acid; (5) Catechin; (6) B-type (epi)catechin trimer; (7) Epicatechin; (8) Coumaroylquinic acid; (9) procyanidin C1; (10) Rutin; (11) Hyperoside; (12) Quercitrin; (13) Isorhamnetin-3-*O*-glucoside; (14) Quercetin.

Figure 5 shows the chromatogram of the isolated TPPs. The identification of individual polyphenolic compounds was accomplished by comparing the characteristic peaks of secondary mass spectra (MS2) and the retention time of parent ions with mixed standards. Table 3 lists the 14 polyphenolic compounds found in the isolated TPPs. The most abundant compounds were chlorogenic acid (120.87 ± 12.09 mg/g of extract), catechin (70.37 ± 4.14 mg/g of extract), and neochlorogenic acid (34.27 ± 1.27 mg/g of extract).

### 3.6. Antioxidant Capacity of TPPs

**Figure 6:**
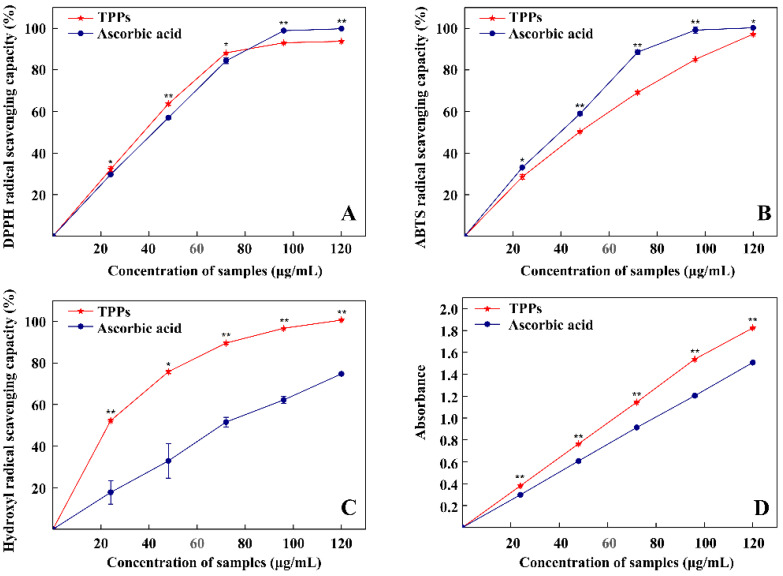
Antioxidant capacity of TPPs: DPPH (**A**), ABTS (**B**), Hydroxyl radical (**C**), FRAP (**D**). * Indicates difference is significant (*p* < 0.05), ** indicates extremely significant (*p* < 0.01).

As shown in Figure 6, TPPs were effective in scavenging DPPH, ABTS, hydroxyl radicals, and ferric ions (measured as FRAP). These antioxidant abilities correlated positively with concentration. TPPs at low concentrations were more effective (*p* < 0.05) than ascorbic acid, but significantly (*p* < 0.01) less effective than when present at 100–120 μg/mL (Figure 6A). Meanwhile, the EC_50_ of TPPs and ascorbic acid were 34.16 ± 0.58 μg/mL and 36.67 ± 0.08 μg/mL, respectively. TPPs showed significantly (*p* < 0.01) lower scavenging effects on ABTS radicals than ascorbic acid, but both had similar scavenging abilities of nearly 100% at a concentration of 120 μg/mL (Figure 6B), and the EC_50_ of TPPs and ascorbic acid were 42.34 ± 0.76 μg/mL and 34.72 ± 0.47 μg/mL, respectively. The hydroxyl radical scavenging capacity (EC_50_ of TPPs was 23.99 ± 0.34 μg/mL, EC50 of ascorbic acid was 68.08 ± 1.04 μg/mL) and FRAP for TPPs were significantly (*p* < 0.01) stronger than for ascorbic acid at the same concentration (Figure 6C,D). These results indicate that TPPs exhibit strong antioxidant properties. In particular, hydroxyl radical scavenging capacity and FRAP ability exceed that exhibited by ascorbic acid.

### 3.7. Inhibition of α-Amylase and α-Glucosidase by TPPs

**Figure 7:**
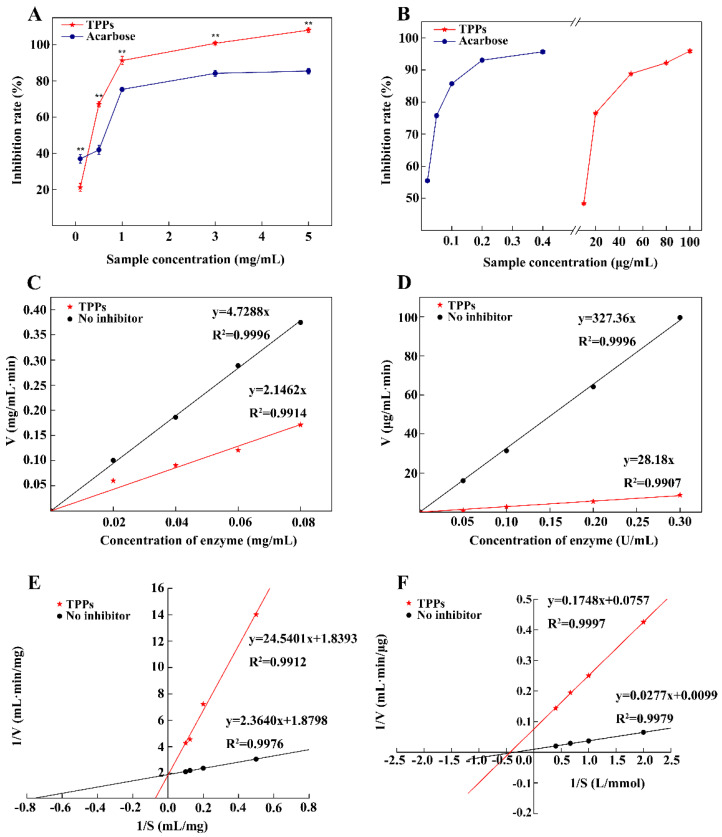
Inhibitory effects (**A**,**B**), inhibition kinetic curve (**C**,**D**) and inhibition modes (**E**,**F**) of TPPs against α-amylase and α-glucosidase. ** indicates extremely significant (*p* < 0.01).

Figure 7A shows that higher concentrations of TPPs have stronger inhibitory effects on a-amylase activity. The inhibition is significantly (*p* < 0.01) higher than that exhibited by acarbose except at low concentration. The EC_50_ of TPPs and acarbose were 0.26 ± 0.02 mg/mL and 0.32 ± 0.03 mg/mL, respectively. The inhibition kinetic curves for a-amylase by TPPs show that velocity and enzyme concentration can be modeled by a straight line through the origin and that the slope of the line associated with TPPs is less than that for the uninhibited control, indicating that TPPs interfere with a-amylase via reversible inhibition (Figure 7C). Further analysis using Lineweaver–Burk double reciprocal curves (Figure 7E) show that the no inhibitor group and the TPPs group intersect on the vertical axis, indicating that the inhibition exerted by TPPs on a-amylase is competitive. In the no inhibitor group, V_max_ = 0.53 mg/(mL·min) and K_m_ = 1.25 mg/mL, whereas in the TPPs group, V_max_ = 0.54 mg/(mL·min) and K_m_ = 13.34 mg/mL. There is no difference in the V_max_ values, but the K_m_ of TPPs is higher, consistent with the characteristics of competitive inhibition (Figure 7E).

Figure 7B shows that TPPs far less effectively inhibit α-glucosidase than acarbose, the positive control. Meanwhile, the EC_50_ of TPPs and acarbose were 9.61 ± 0.05 μg/mL and 0.02 ± 0.01 μg/mL, respectively. Moreover, the inhibition kinetic profile for α-glucosidase is similar to that of α-amylase, illustrating that the mode of inhibition of α-glucosidase by TPPs is also reversible (Figure 7D). The Lineweaver–Burk double reciprocal curves for α-glucosidase intersect on the abscissa. In the no inhibitor group, V_max_ = 101.01 μg/(mL·min), and K_m_ = 2.80 mmol/L, while in the TPPs group, V_max_ = 13.21 μg/(mL·min) and K_m_ = 2.31 mmol/L. These results demonstrate that the reversible inhibition of TPPs against α-glucosidase can be classified as noncompetitive (Figure 7F). Thus, TPPs have high hypoglycemic ability in vitro, and the inhibitory effect on a-amylase activity exceeds that of acarbose.

### 3.8. Binding of Bile Acid by TPPs

**Figure 8:**
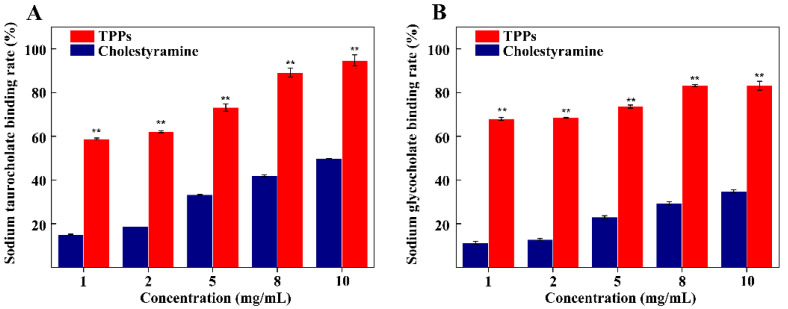
Bile acid binding capacity of TPPs. (**A**) Sodium taurocholate binding capacity, (**B**) sodium glycocholate binding capacity. ** Indicates extremely significant (*p* < 0.01).

TPPs concentration and binding rate are strongly correlated. The sodium taurocholate binding rate spans 59–95% at concentrations from 1 to 10 mg/mL, and in all cases the binding is significantly (*p* < 0.01) stronger than that exhibited by choles-tyramine, and the EC_50_ of TPPs and cholestyramine were 0.87 ± 0.03 mg/mL and 11.49 ± 0.06 mg/mL, respectively (Figure 8A). TPPs also have a similar binding capacity for sodium glycocholate as for sodium taurocholate (Figure 8B). The EC_50_ of TPPs and cholestyramine were 0.19 ± 0.03 mg/mL and 28.39 ± 1.81 mg/mL, respectively. At concentrations from 1 to 10 mg/mL, the binding rate of TPPs is 68 to 83%, and the binding effect is stronger than that of cholestyramine (*p* < 0.01). It is noteworthy that the binding rate of TPPs to bile acid is more than twice that of cholestyramine at the same concentration. Thus, TPPs have a higher binding capacity for sodium taurocholate/sodium taurocholate and thus a higher hypolipidemic effect in vitro than does the positive control (cholestyramine).

## 4. Discussion

Although polyphenols in thinned apples, pears, and grapes have attracted interest, relatively few investigations have focused on TPPs [2]. These studies tend to emphasize polyphenol composition and function, while polyphenol extraction and purification have not received the same attention [8,9]. Yue et al. [27] found that ultrasonic power, extraction time, and temperature has significant effects on the extraction of thinned apple polyphenols. Compared with solvent extraction, the biggest advantages of ultrasonic assisted polyphenol extraction are reduced extraction time and increased yield. For example, for the extraction of polyphenols from thinned apple, a 6 h solvent extraction yielded 13.96 mg GAE/g DW (drying weight). In contrast, ultrasonic assisted extraction for 1 h yielded 32.63 mg GAE/g DW [28]. In our study using thinned peaches, TPPs were also extracted using an ultrasonic-assisted protocol. The results were consistent with those obtained Yue et al. [27], but with an extraction time of only 25 min. The yield of TPPs reached 1.59 mg GAE/g FW (about 7.95 mg GAE/g DW), lower than that from thinned apple and thinned pear (16.4 mg GAE/g FW) [6], but higher than that from thinned areca (1.5 mg GAE/g FW) [29], thinned apricot (1.2 mg GAE/g FW) [8], thinned nectarine (1.19 mg GAE/g FW) [8], and thinned mulberry (0.76 mg GAE/g FW) [30].

Macroporous resins have been widely used to separate and purify polyphenols because of their unique advantages, including specific adsorption capacity provided by non-covalent bonds and aromatic accumulation, low cost, and easy resin regeneration [12,31]. Sun et al. [32] used X-5 and polyamide resin to obtain polyphenol monomers from thinned apple, including chlorogenic acid, epicatechin, hyperoside, and phlorizin. Sun et al. [33] purified thinned apple polyphenols with X-5 resin, increasing purity 2.12-fold from 35.17% to 74.64%. Factors such as pH, ethanol concentration, and flow rate can affect the purification of thinned apple polyphenols [33]. We also identified other important factors that affect purification. For example, we found that purification was improved by performing extraction at pH 3, with a sample concentration of 1 mg/mL, an adsorption speed of 1 mL/min, a desorption concentration of 50%, a desorption speed of 1 mL/min, a loading volume of 1.5 BV, and an elution volume of 6 BV. NKA-9 resin had the best performance for purification. Overall, the optimized protocol increased concentration from 1.93% to 43.86%. It is important to note that the biological activities of polyphenols after purification were higher than the activities measured using crude extracts. Yang et al. [10] also found that purified polyphenol obtained from unripe raspberry fruits was more active than the unpurified extract in terms of antioxidant capacity and antibacterial activity.

In their study of the composition of TPPs, Guo et al. [3] found that neochlorogenic acid, catechin, and chlorogenic acid were the primary polyphenols in seven different thinned peach varieties, and that the varieties could be distinguished based on their phenolic acids. Guo et al. [9] also reported that the metabolic markers neochlorogenic acid, catechin, and chlorogenic acid distinguished unripe and mature peaches, and that the proportions of these three components in thinned peach varieties tended to be neochlorogenic acid > catechin > chlorogenic acid. In our study, the main components of TTPs from cv. Hujingmilu were chlorogenic acid, catechin, and neochlorogenic acid, with the proportions ranked as chlorogenic acid > catechin > neochlorogenic acid.

Polyphenols have diverse biological activities, including antioxidant, hypoglycemic, and hypolipidemic effects [2]. In order to systematically explore and evaluate the antioxidant capacity of TPPs, it is advantageous to test multiple indicators simultaneously [34]. Guo et al. [9] evaluated the antioxidant capacity of peach polyphenols from thinned fruit against DPPH radicals, ABTS radicals, and FRAP, and found that the antioxidant capacity was 1.3–11.2 times higher than for polyphenols recovered from mature fruit. Similarly, thinned apple polyphenols exhibit scavenging capacity for DPPH [27], and there is a high correlation between polyphenol content and antioxidant capacity in thinned pear [6]. In this study, TPPs were found to have high scavenging capacity against DPPH radicals, ABTS radicals, hydroxyl radicals, and FRAP. Liu et al. [15] reported that the EC_50_ of polyphenol crude extracts obtained from cv. Hujingmilu peach flesh and peels was 60 mg/mL and 30 mg/mL against DPPH radicals, respectively. In contrast, the EC_50_ of purified TPPs in our study was only 40 μg/mL. We conclude that the TPPs we isolated have a markedly superior antioxidant capacity.

α-Amylase and α-glucosidase are key digestive enzymes that accelerate the decomposition of starch, contributing to the elevation of blood sugar. Polyphenols have the ability to chelate digestive enzymes [18]. Sun et al. [35] found that thinned apple polyphenols had high inhibitory activity against α-amylase, and the mode of inhibition was competitive. Chen et al. [36] also showed that chingiitannin A from the unripe fruit of *Rubus chingii* Hu had the highest inhibitory activities against α-amylase and α-glucosidase, and had a reversible and noncompetitive mode of inhibition. Mihaylova et al. [37] found that crude polyphenol extracts from eight peach varieties had no inhibitory effect on α-amylase, but the TPPs identified in our study inhibit both α-amylase and α-glucosidase, via competitive reversible inhibition and noncompetitive reversible inhibition. In addition, the EC_50_ of TPPs to α-amylase was only 0.26 mg/mL, making it potentially useful for the control of blood sugar.

In humans, hyperlipidemia is closely related to bile acid. Polyphenols can prevent the reabsorption of bile acid in the small intestine by combining with bile acid, thus reducing hyperlipidemia [19]. In our study, TPPs showed higher binding than cholestyramine on the common bile acids sodium taurocholate and sodium glycocholate. With the exception of Joymak et al. [38], we have not found any studies on related functions in the published literature on thinned fruits. Joymak et al. [38] found that the binding rate of polyphenols from unripe papaya was about 8% when the concentration was 2 mg/mL. In contrast, we found that the binding rate of TPPs from thinned peaches to bile acid can reach more than 60% at the same concentration, a level at which TPPs are potentially useful for the treatment of hyperlipidemia.

## 5. Conclusions

In this study, we optimized the extraction and purification process for TPPs from thinned peaches. The yield was 1.59 ± 0.02 mg GAE/g FW (about 7.95 mg GAE/g DW) and the purity was 43.86%. Although the content of TPPs was not high, thinned peaches are abundant, and thus deserve attention as a potentially valuable resource. We found that chlorogenic acid, catechin, and neochlorogenic acid were the primary polyphenols in thinned peaches. These components may be responsible for the higher biological activity exhibited by TPPs than by our positive control, including the significantly higher capacity for scavenging hydroxyl radicals and FRAP, the inhibition of α-amylase, and the binding of bile acid. These properties suggest that TPPs may be useful for the improvement of human health. Therefore, our results provide a basis for the development and utilization of thinned peaches.

## Figures and Tables

**Table 1:** Design and results of Box–Behnken response surface experiment.

Number	Ultrasonic Time (min)	Ultrasonic Temperature (°C)	Ultrasonic Power (W)	Solid-to-Liquid (g/mL)	Yield of Polyphenols (mg GAE/g FW)
1	10 (−1)	30 (−1)	150 (0)	1:10 (0)	1.331 ± 0.026
2	30 (1)	30 (−1)	150 (0)	1:10 (0)	1.152 ± 0.008
3	10 (−1)	50 (1)	150 (0)	1:10 (0)	1.351 ± 0.010
4	30 (1)	50 (1)	150 (0)	1:10 (0)	1.389 ± 0.015
5	20 (0)	40 (0)	120 (−1)	1:8 (−1)	1.066 ± 0.027
6	20 (0)	40 (0)	180 (1)	1:8 (−1)	1.113 ± 0.014
7	20 (0)	40 (0)	120 (−1)	1:12 (1)	1.370 ± 0.030
8	20 (0)	40 (0)	180 (1)	1:12 (1)	1.489 ± 0.022
9	10 (−1)	40 (0)	150 (0)	1:8 (−1)	1.137 ± 0.039
10	30 (1)	40 (0)	180 (1)	1:8 (−1)	1.077 ± 0.021
11	10 (−1)	40 (0)	150 (0)	1:12 (1)	1.449 ± 0.009
12	30 (1)	40 (0)	150 (0)	1:12 (1)	1.489 ± 0.016
13	20 (0)	30 (−1)	120 (−1)	1:10 (0)	1.181 ± 0.009
14	20 (0)	50 (1)	120 (−1)	1:10 (0)	1.354 ± 0.060
15	20 (0)	30 (−1)	180 (1)	1:10 (0)	1.246 ± 0.075
16	20 (0)	50 (1)	180 (1)	1:10 (0)	1.316 ± 0.036
17	10 (−1)	40 (0)	120 (−1)	1:10 (0)	1.214 ± 0.029
18	30 (1)	40 (0)	120 (−1)	1:10 (0)	1.297 ± 0.033
19	10 (−1)	40 (0)	180 (1)	1:10 (0)	1.287 ± 0.015
20	30 (1)	40 (0)	180 (1)	1:10 (0)	1.187 ± 0.019
21	20 (0)	30 (−1)	150 (0)	1:8 (−1)	1.087 ± 0.012
22	20 (0)	50 (1)	150 (0)	1:8 (−1)	1.232 ± 0.005
23	20 (0)	30 (−1)	150 (0)	1:12 (1)	1.485 ± 0.040
24	20 (0)	50 (1)	150 (0)	1:12 (1)	1.547 ± 0.041
25	20 (0)	40 (0)	150 (0)	1:10 (0)	1.424 ± 0.040
26	20 (0)	40 (0)	150 (0)	1:10 (0)	1.419 ± 0.043
27	20 (0)	40 (0)	150 (0)	1:10 (0)	1.408 ± 0.040
28	20 (0)	40 (0)	150 (0)	1:10 (0)	1.386 ± 0.041
29	20 (0)	40 (0)	150 (0)	1:10 (0)	1.399 ± 0.013

**Table 2:** Results of ANOVA from response surface experiment.

Source	Sum of Squares	df	Mean Square	F-Value	*p*-Value	Significant
Model	0.53	14	0.038	53.42	<0.0001	**
A (ultrasonic time)	2.61 × 10^−3^	1	2.61 × 10^−3^	3.68	0.0757	
B (ultrasonic temperature)	0.042	1	0.042	58.77	<0.0001	**
C (ultrasonic power)	2.00 × 10^−3^	1	2.00 × 10^−3^	2.82	0.1152	
D (solid-to-liquid)	0.37	1	0.37	525.61	<0.0001	**
AB	0.012	1	0.012	16.61	0.0011	**
AC	8.45 × 10^−3^	1	8.45 × 10^−3^	11.89	0.0039	**
AD	2.51 × 10^−3^	1	2.51 × 10^−3^	3.54	0.081	
BC	2.68 × 10^−3^	1	2.68 × 10^−3^	3.78	0.0723	
BD	1.69 × 10^−3^	1	1.69 × 10^−3^	2.38	0.1453	
CD	1.28 × 10^−3^	1	1.28 × 10^−3^	1.8	0.2011	
A^2^	0.031	1	0.031	43.34	<0.0001	**
B^2^	5.89 × 10^−3^	1	5.89 × 10^−3^	8.29	0.0121	*
C^2^	0.063	1	0.063	89.3	<0.0001	**
D^2^	0.014	1	0.014	19.68	0.0006	**
Residual	9.95 × 10^−3^	14	7.10 × 10^−3^			
Lack of Fit	8.99 × 10^−3^	10	8.99 × 10^−3^	3.76	0.1069	
Cor Total	0.54	28				
R^2^	0.9816					
R_adj_^2^	0.9632					

Note: * indicates (*p* < 0.05), ** (*p* < 0.01).

**Table 3:** List of identified TPPs.

Peak	RT (min)	MW	MS (m/z)	MS/MS (m/z)	Tentative Identification	Molecular Formula
1	6.015	354	355	135	Neochlorogenic acid [20]	C_16_H_18_O_9_
2	7.355	449	450	287	Cyanidin-3-glucoside [21]	C_21_H_21_O_11_
3	7.715	578	579	289	Procyanidin B1 [21]	C_30_H_27_O_12_
4	7.842	354	355	135	Chlorogenic acid [20]	C_16_H_18_O_9_
5	7.975	290	291	139, 123	Catechin [22]	C_15_H_14_O_6_
6	8.302	866	867	289, 287	B-type (epi)catechin trimer [22]	C_45_H_38_O_18_
7	9.035	290	291	139, 123	Epicatechin [22]	C_15_H_14_O_6_
8	9.155	338	339	119	Coumaroylquinic acid [20]	C_16_H_18_O_8_
9	9.582	866	867	451, 289	procyanidin C1 [23]	C_45_H_38_O_18_
10	10.502	610	611	303	Rutin [24]	C_27_H_30_O_16_
11	10.889	464	465	303	Hyperoside [24]	C_21_H_20_O_12_
12	11.349	448	449	303	Quercitrin [24]	C_21_H_20_O_11_
13	11.715	478	479	151	Isorhamnetin-3-*O*-glucoside [25]	C_22_H_22_O_12_
14	14.229	302	303	257, 153	Quercetin [26]	C_15_H_10_O_7_

## Data Availability

The data supporting the findings of this study are included within the article.

## Supplementary Materials

The following supporting information can be downloaded at: https://www.mdpi.com/article/10.3390/foods11010099/s1, Figure S1: Standard curves for sodium taurocholate (A) and sodium glycocholate (B), Table S1: Independent factors and levels in the response surface experiment, Table S2: Physical characteristics of the four macroporous resins, Table S3: Linear equations, linear ranges and correlation coefficients for each component of mixed standard.
