# Supplementary material for: Lab Scale Extracted Conditions of Polyphenols from Thinned Peach Fruit Have Antioxidant, Hypoglycemic, and Hypolipidemic Properties
Source: Foods. 2021 Dec 31;11(1):99. doi: 10.3390/foods11010099 (PMC8750482; doi:10.3390/foods11010099)
Supplement: Supplementary file 1 [file foods-11-00099-s001.zip › foods-1510204-supplementary.pdf]

Supplementary Materials

Table S1. Independent factors and levels in the response surface experiment.

Factors	Code	Level		
		-1	0	1
Ultrasonic time (min)	A	10	20	30
Ultrasonic temperature (°C)	B	30	40	50
Ultrasonic power (W)	C	120	150	180
Solid -to-liquid (g/mL)	D	1:8	1:10	1:12

Table S2. Physical characteristics of the four macroporous resins.

Resins	Specific surface area（m^2/g）	Average pore diameter（nm）	Polarity
NKA-9	500-550	10-12	Polar
AB-8	480-520	13-14	Weak polar
D101	550-600	9-10	Nonpolar
X-5	500-650	28-30	Nonpolar

Table S3. Linear equations, linear ranges and correlation coefficients for each component of mixed standard.

Analytes	Regression equation	Linear range (μg/mL)	R^2
Neochlorogenic acid	y=10.1159x+23.0507	10-250	0.9994
Chlorogenic acid	y=10.3652x-77.8026	10-250	0.9987
Catechin	y=4.7892x-5.5950	10-500	0.9999

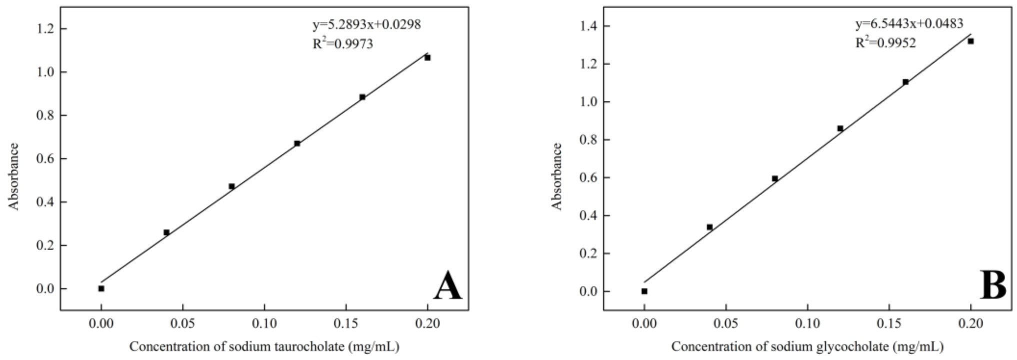

Figure S1. Standard curves for sodium taurocholate (A) and sodium glycocholate (B).
